# Supplementary material for: FourCSeq: analysis of 4C sequencing data
Source: Bioinformatics. 2015 Jun 1;31(19):3085–91. doi: 10.1093/bioinformatics/btv335 (PMC4576695; doi:10.1093/bioinformatics/btv335)
Supplement: Supplementary Data [file supp_31_19_3085__index.html]

FourCSeq: analysis of 4C sequencing data — FourCSeq: analysis of 4C sequencing data — Supplementary Data 

# FourCSeq: analysis of 4C sequencing data

## Supplementary Data

files

- Supplementary Data - pdf file
