# Supplementary material for: FourCSeq: analysis of 4C sequencing data
Source: Bioinformatics. 2015 Jun 1;31(19):3085–91. doi: 10.1093/bioinformatics/btv335 (PMC4576695; doi:10.1093/bioinformatics/btv335)
Supplement: Supplementary Data [file supp_btv335_Supplement_File_1.pdf]

Supplementary Information:
FourCSeq comparison with r3Cseq

Felix A. Klein, Tibor Pakozdi, Simon Anders, Yad Ghavi-Helm, Eileen E. M. Furlong, Wolfgang Huber
European Molecular Biology Laboratory (EMBL),
Heidelberg, Germany
felix.klein@embl.de

May 8, 2015

Contents

1 Analysis of 4C data from the Myb promoter **1**
- 1.1 r3Cseq Analysis . . . 1
- 1.2 FourCSeq Analysis . . . 3
- 1.3 Result of the comparison . . . 9

2 Analysis of 4C data from the Tfap2c viewpoint **9**
- 2.1 r3Cseq analysis . . . 9
- 2.2 FourCSeq Analysis . . . 10
- 2.3 Result of the comparison . . . 14

Load the required packages.

```
library("FourCSeq")
library("r3Cseq")

## Warning in fun(libname, pkgname): couldn't connect to display ":0"

library("BSgenome.Mmusculus.UCSC.mm9")
```

1 Analysis of 4C data from the Myb promoter

For a comparison of *FourCSeq* with *r3Cseq* we use the 4C seq data set of the Myb promoter provided by the authors of *r3Cseq* on their website http://r3cseq.genereg.net/Site/index.html.

1.1 r3Cseq Analysis

First we run the r3Cseq pipeline as described on on the r3Cseq website http://r3cseq.genereg.net/Site/index.html

First the `r3Cseq` object is created.

```
infiles <- c("Myb_prom_FB_rep1.bam", "Myb_prom_FB_rep2.bam",
             "Myb_prom_FL_rep1.bam", "Myb_prom_FL_rep2.bam")

myBatch_obj <- new("r3CseqInBatch",
                   organismName = 'mm9',
                   restrictionEnzyme = 'HindIII',
                   isControlInvolved = TRUE,
                   bamFilesDirectory = ".",
```

```
                    viewpoint_chromosome = 'chr10',
                    viewpoint_primer_forward = 'TCTTTGTTTGATGGCATCTGTT',
                    viewpoint_primer_reverse = 'AAAGGGGAGGAGAAGGAGGT',
                    BamExpFiles = infiles[1:2],
                    BamContrFiles = infiles[3:4],
                    expBatchLabel = c("Myb_FL1", "Myb_FL2"),
                    contrBatchLabel = c("Myb_FB1", "Myb_FB2"))
```

Next the read bam files are read and reads mapping to restriction fragments are counted.

```
getBatchRawReads(myBatch_obj)

## [1] "start reading in... Myb_prom_FB_rep1.bam ....file in the experiment"
## [1] "start reading in... Myb_prom_FB_rep2.bam ....file in the experiment"
## [1] "start reading in... Myb_prom_FL_rep1.bam ....file in the control"
## [1] "start reading in... Myb_prom_FL_rep2.bam ....file in the control"
## [1] "The raw read files are created!!!."

getBatchReadCountPerRestrictionFragment(myBatch_obj)

## [1] "Fragmenting genome.........."
## [1] "start counting reads in... Myb_prom_FB_rep1.bam ....file in the experiment"
## [1] "start counting reads in... Myb_prom_FB_rep2.bam ....file in the experiment"
## [1] "start counting reads in... Myb_prom_FL_rep1.bam ....file in the control"
## [1] "start counting reads in... Myb_prom_FL_rep2.bam ....file in the control"
## [1] "The countTable is created!!!."
```

The data is then normalized and RPM are calculated for each fragment.

```
calculateBatchRPM(myBatch_obj)

## [1] "Calculating RPMs reads in... Myb_prom_FB_rep1.bam ....file in the experiment"
## [1] "Calculating RPMs reads in... Myb_prom_FB_rep2.bam ....file in the experiment"
## [1] "Calculating RPMs reads in... Myb_prom_FL_rep1.bam ....file in the control"
## [1] "Calculating RPMs reads in... Myb_prom_FL_rep2.bam ....file in the control"
## [1] "The RPMs table is created!!!."
```

After normalization, interactions are calculated using the conservative method "intersection", which only takes the intersection of interactions identified in the two replicates per viewpoint.

```
getBatchInteractions(myBatch_obj, method = "intersection")

## [1] "Analyzing interaction regions ... Myb_prom_FB_rep1.bam .... in the experiment"
## [1] "Analyzing interaction regions ... Myb_prom_FB_rep2.bam .... in the experiment"
## [1] "Analyzing interaction regions ... Myb_prom_FL_rep1.bam .... in the control"
## [1] "Analyzing interaction regions ... Myb_prom_FL_rep2.bam .... in the control"
## [1] "Interactions from the intersection method are created!!!."
```

The results are visualized with the provided plotting functions.

```
plotInteractionsNearViewpoint (myBatch_obj)
```

```
plotInteractionsPerChromosome(myBatch_obj, "chr10")
```

```
plotOverviewInteractions(myBatch_obj)
```

Figure 1 shows the 4C signal in a 500 kb window around the viewpoint. Interactions are defined by setting a threshold on the q-values calculated for each fragment. Levels of q-values are represented by colored points for the experiment (red) and control (blue). Several fragments with low q-values are identified as interactions in this case.

Figure 2 shows an overview of all interactions on the viewpoint chromosome. Interactions cluster in close proximity to the viewpoint.

A global overview of interactions on all chromosomes is shown in Figure 3. The major of interaction calls are made on the viewpoint chromosome.

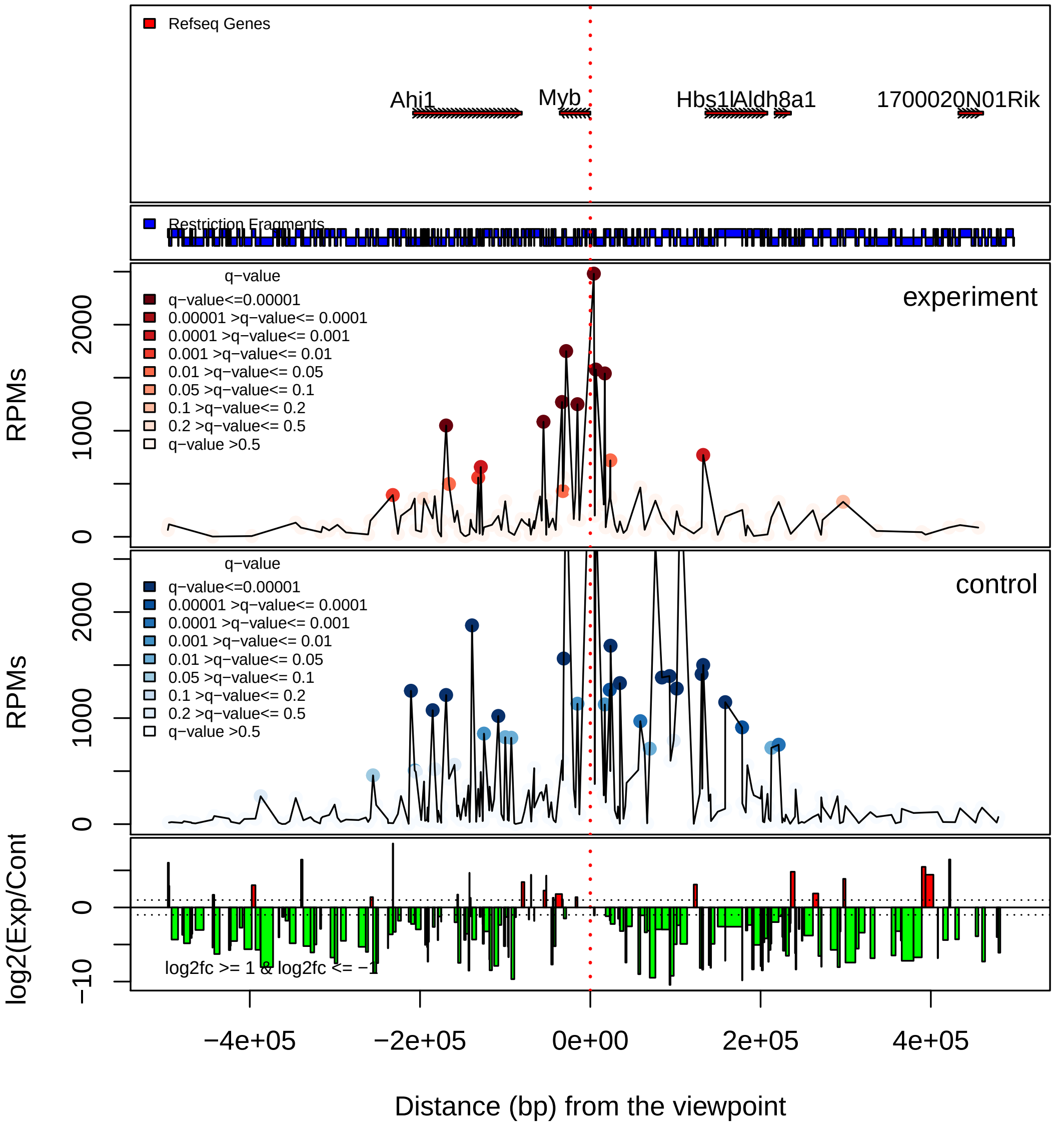

Figure 1: r3Cseq: Overview plot of interactions near the viewpoint.

1.2 FourCSeq Analysis

Now we run the analysis using *FourCSeq*. First we prepare the *FourC* object.

```
projectPath = "FourCSeq"
exptData <- SimpleList(projectPath=projectPath,
                       dataDir="filtered_coverage",
                       fragmentDir="re_fragments",
                       referenceGenomeFile=BSgenome.Mmusculus.UCSC.mm9,
                       reSequence1="AAGCTT",
                       reSequence2="CATG",
                       primerFile =  "primers.fa",
                       bamFilePath=".")

tmp <- strsplit(infiles, "_")
tmpLength <- sapply(tmp, length)
vp <- sapply(tmp, "[", 1)
condition <- sapply(tmp, "[", 3)
replicate <- gsub("rep|.bam", "", sapply(tmp, "[", 4))
```

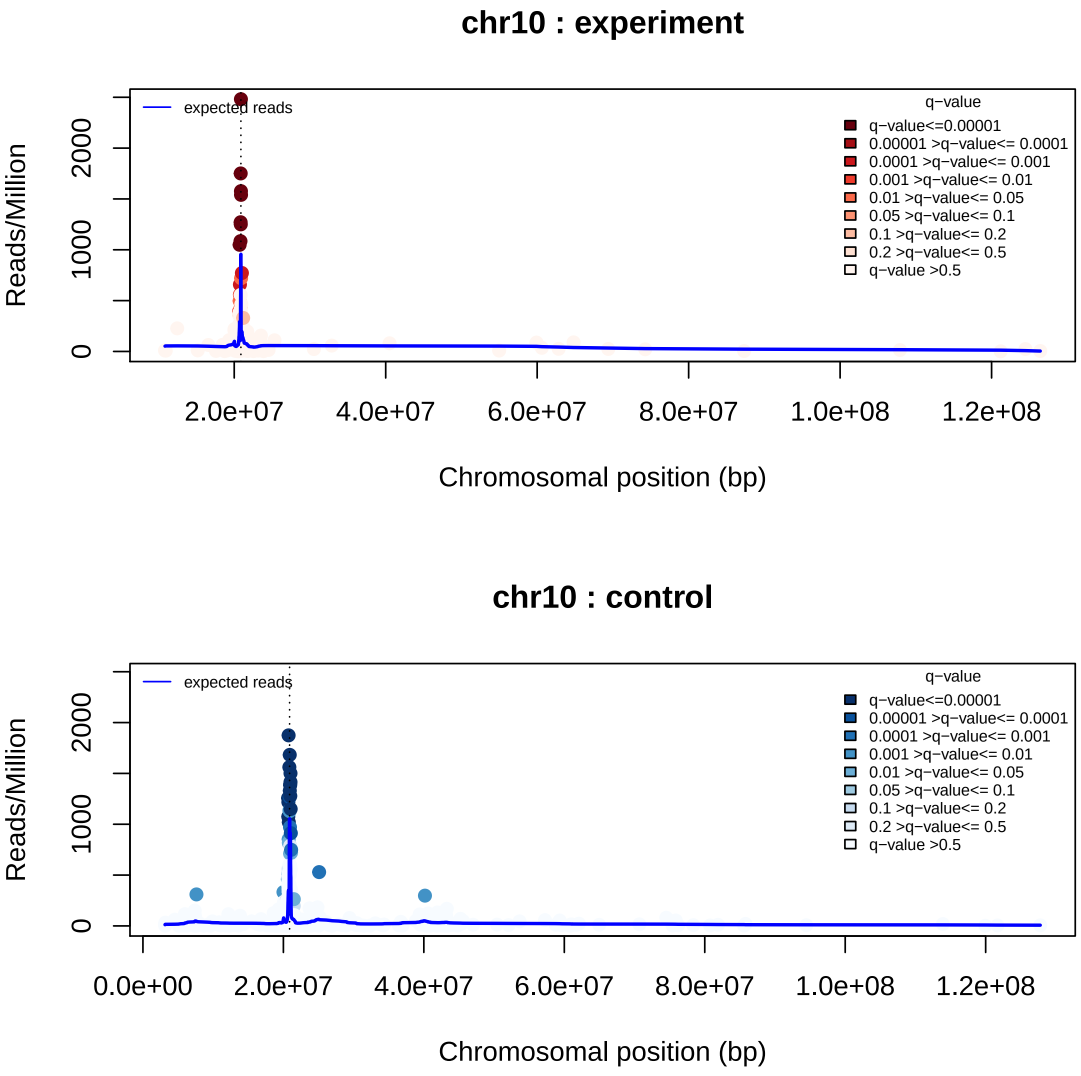

Figure 2: r3Cseq: Overview plot of all interactions on the viewpoint chromosome.

```
rm("tmp")

colData <- DataFrame(viewpoint = vp,
                     condition = condition,
                     replicate= replicate,
                     bamFile = infiles,
                     sequencingPrimer = "first")

fc <- FourC(colData, exptData)
fc

## class: FourC
## dim: 0 4
## exptData(8): projectPath dataDir ... primerFile bamFilePath
## assays(0):
## rownames: NULL
## rowData metadata column names(0):
## colnames(4): Myb_FB_1 Myb_FB_2 Myb_FL_1 Myb_FL_2
```

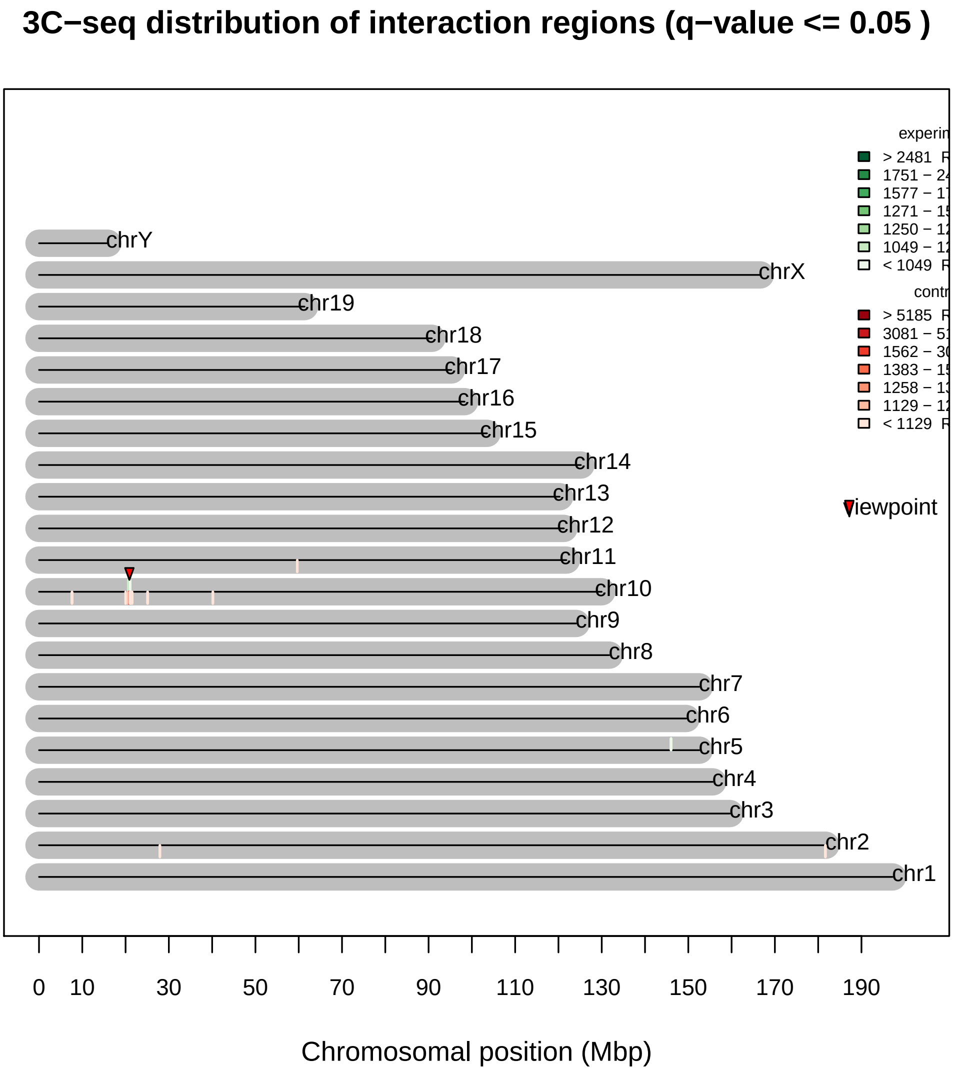

Figure 3: r3Cseq: Overview plot of interactions for all chromosomes.

```
## colData names(5): viewpoint condition replicate bamFile sequencingPrimer
```

In the next step the reference of restriction fragments is generated and the position of the viewpoint and the viewpoint fragment are identified using the viewpoint primer sequence.

```
fc <- addFragments(fc, filter=FALSE)
findViewpointFragments(fc)
fc <- addViewpointFrags(fc)
```

After creating a corresponding `FourC` object with the reference fragments the number of reads overlapping with fragment ends are counted.

```
fc <- countFragmentOverlaps(fc, shift=5, minMapq=-1)

## reading bam files
## calculating overlaps

fc <- combineFragEnds(fc)
```

Now we use the `getZScores` function to detect interactions.

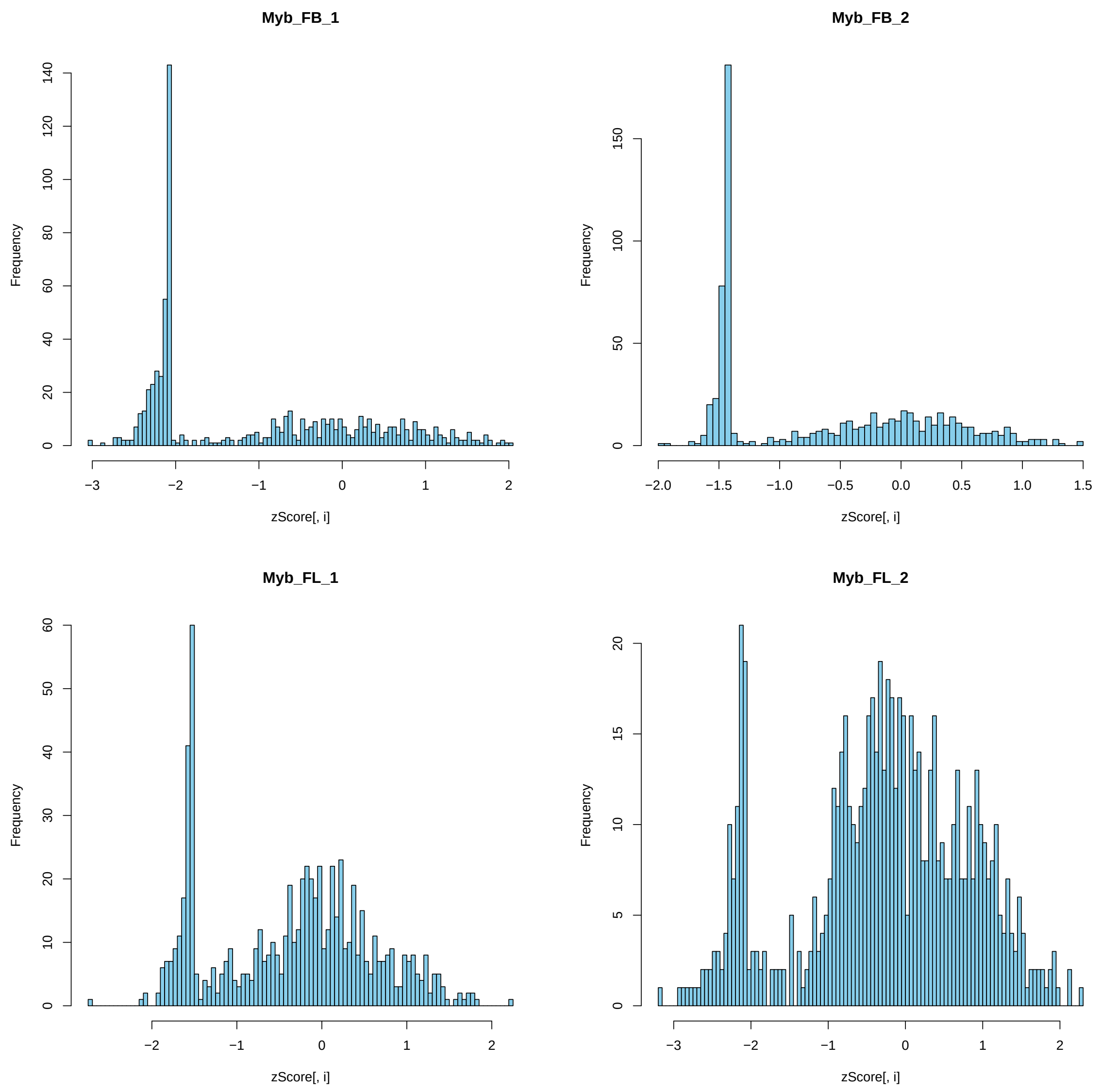

Figure 4: Histograms of z-scores for the different replicates

```
fcf <- getZScores(fc)
```

We take a look at the distribution of z-scores, which are stored in the assay element `zScore` of the `FourC` object.

```
zScore <- assay(fcf, "zScore")

for(i in seq_len(ncol(zScore)))
    hist(zScore[,i], breaks = 100, main=colnames(zScore)[i], col="skyblue")
```

Figure 4 shows that in all cases there is a side peak (at positions ∼ -1.5 to -2) on the left. This is due to fragments that have 0 reads in one library and many reads in another. The unusual shape of the z-score distributions could already alert the user of a data quality problen, i.e., in this case, high variability between replicates due to apparently stochastic PCR amplification events.

Interactions are annotated using the `addPeaks` function after the z-scores are calculated.

```
fcf <- addPeaks(fcf)
```

Next we take a look at the fit for the first sample.

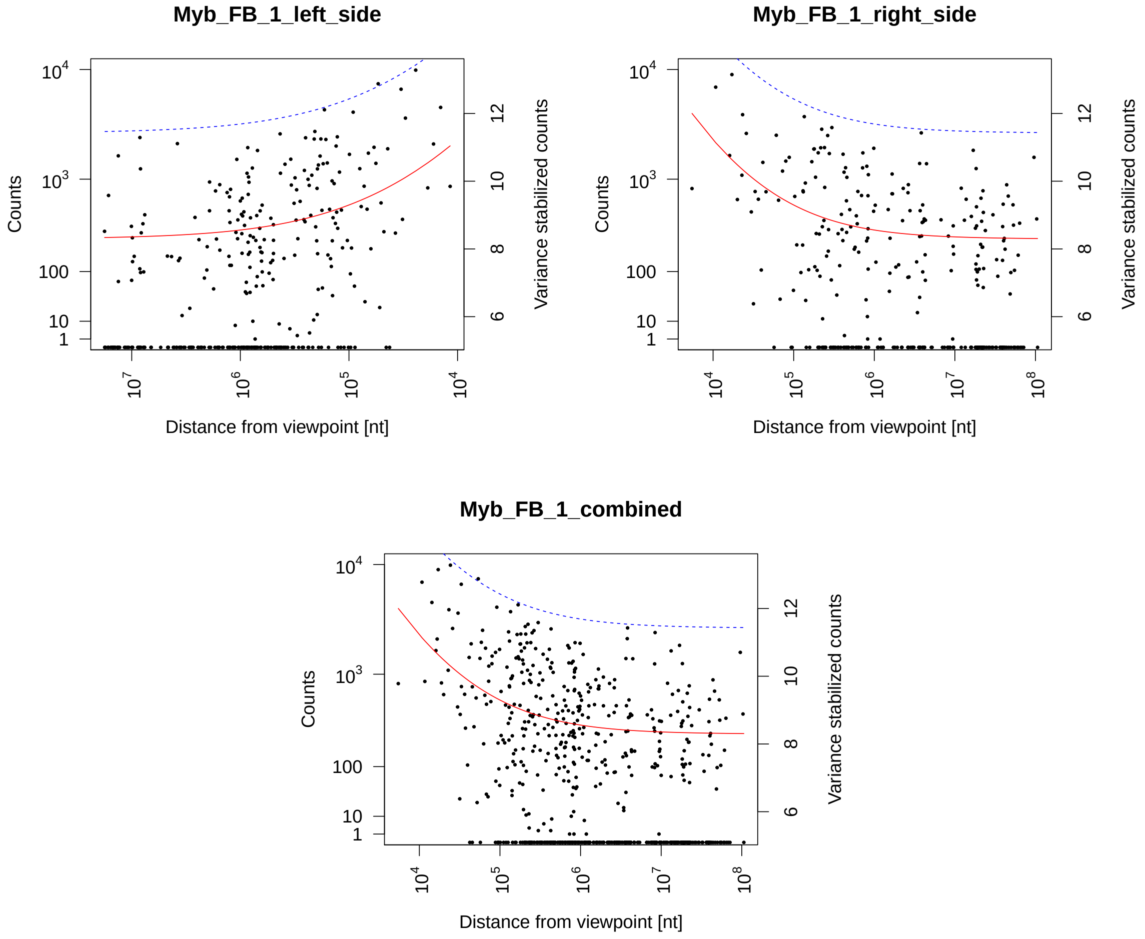

Figure 5: Distance fit on the variance stabilized read counts.

```
plotFits(fcf[,1])
```

Figure 5 shows the fit on the left side of the viewpoint, on the right side, and for both sides combined and ploted over the absolut distance to the viewpoint. The points in Figure 5 show the count values for the individual fragments. The red line is the fit and the blue dashed line is the fit plus (z-score threshold)*MAD for the given library, where the z-score threshold has been defined by the call to `addPeaks`. The first plot show the data left of the viewpoint, the second plot right of the viewpoint and for the last plot both sides have been combined by using the absolute distance from the viewpoint.

The results are visualized in the next step. The transcript annotation from the *TxDb.Mmusculus.UCSC.mm9.knownGene* package is included in the plots.

```
library("TxDb.Mmusculus.UCSC.mm9.knownGene")

plotZScores(fcf,
            plotWindows = 5e+05,
            txdb=TxDb.Mmusculus.UCSC.mm9.knownGene)

## [1] "Myb"
```

```
## Successfully plotted results.
```

The plot in Figure 6 shows the results for a 500 kb window around the viewpoint. The fit is shown as green line and the dashed blue lines span the interval of $\pm$ (z-score threshold)*MAD, where the z-score threshold of 2 has been defined by the call to `addPeaks`. **There are no interactions called in this analysis by the FourCSeq package.**

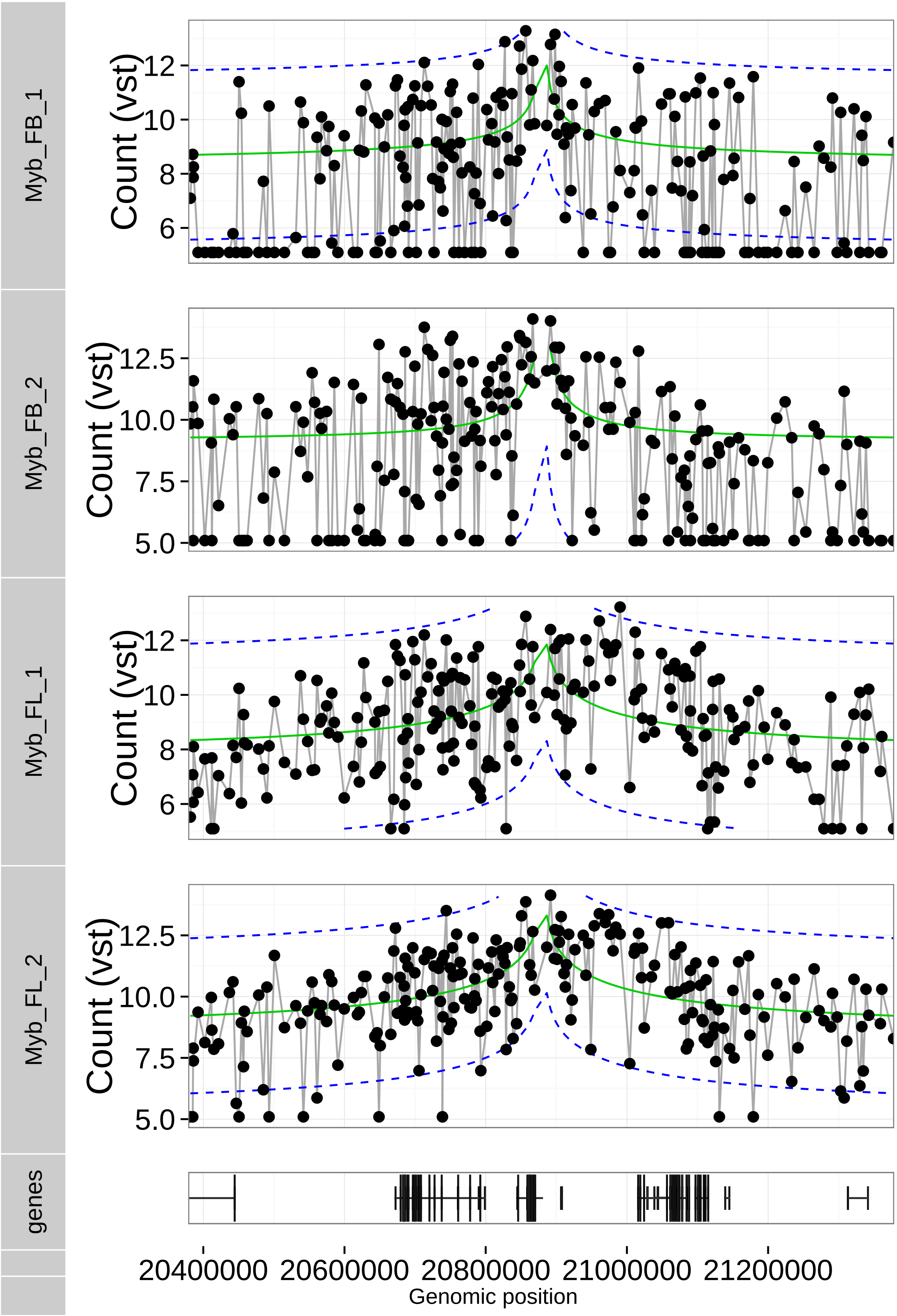

Figure 6: Overview plot of z-scores near to the viewpoint.

1.3 Result of the comparison

For the Myb 4C data our *FourCSeq* analysis does not detect any specific chromatin interactions. This is because the replicates do not show sufficient agreement. The *r3Cseq* however detected many interactions (Figure 1), even with the conservative "intersection" approach. These correspond to the fragments close to the viewpoint which (happen to) have high read counts in both libraries. While we cannot definitely exclude their correctness, arguably, the strength of evidence from the data is not high (because of the PCR duplication artefacts and disagreement between replicates that we identified).

Conclusion: this analysis shows that our *FourCSeq* method does not detect specific interactions, if the underlying data are not consistent. r3Cseq can produce results even in the face of highly divergent replicate data.

2 Analysis of 4C data from the Tfap2c viewpoint

Here we analyse a dataset from [1]. This 4C dataset is of good quality, but unfortunately there are no technical replicates. However, the wild-type samples of the Tfap2c viewpoint for the whole embryo wild-type (wt) and medial forebrain wild-type (wt MFB) sample are very similar and here we treat them as replicates for this analysis comparison. The dataset is available at the ENA (http://www.ebi.ac.uk/ena/data/view/ERP005557).

2.1 r3Cseq analysis

Here we run exactly the same workflow as above for the Myb promoter viewpoint.

```
bamFiles = c("ap2c_wt.bam",
             "ap2c_wtMFB.bam")

my_r3Cseq_obj <- new("r3Cseq",
              organismName = 'mm9',
              restrictionEnzyme = 'NlaIII',
              isControlInvolved=TRUE,
              viewpoint_chromosome = 'chr2',
              viewpoint_primer_forward = 'TCAACAACCCTCCTCCCATG',
              viewpoint_primer_reverse = 'GATAGGCTCACAACGAAGTC',
              alignedReadsBamExpFile = bamFiles[1],
              alignedReadsBamContrFile = bamFiles[2],
              expLabel = c("Tfap2c_wt"),
              contrLabel = c("Tfap2c_MFB"))

getRawReads(my_r3Cseq_obj)

## [1] "start reading in ......"
## [1] "Raw reads processing is done."

getReadCountPerRestrictionFragment(my_r3Cseq_obj)

## [1] "Fragmenting genome by....NlaIII...."
## [1] "Count processing in the experiment is done."
## [1] "Count processing in the control is done."

calculateRPM(my_r3Cseq_obj)

## [1] "Normal RPM calculation is done."

getInteractions(my_r3Cseq_obj)

## [1] "Calculation is done. Use function 'expInteractionRegions' or 'contrInteractionRegions' to get t
```

The results are visualized with the provided plotting functions.

```
plotInteractionsNearViewpoint (my_r3Cseq_obj)
```

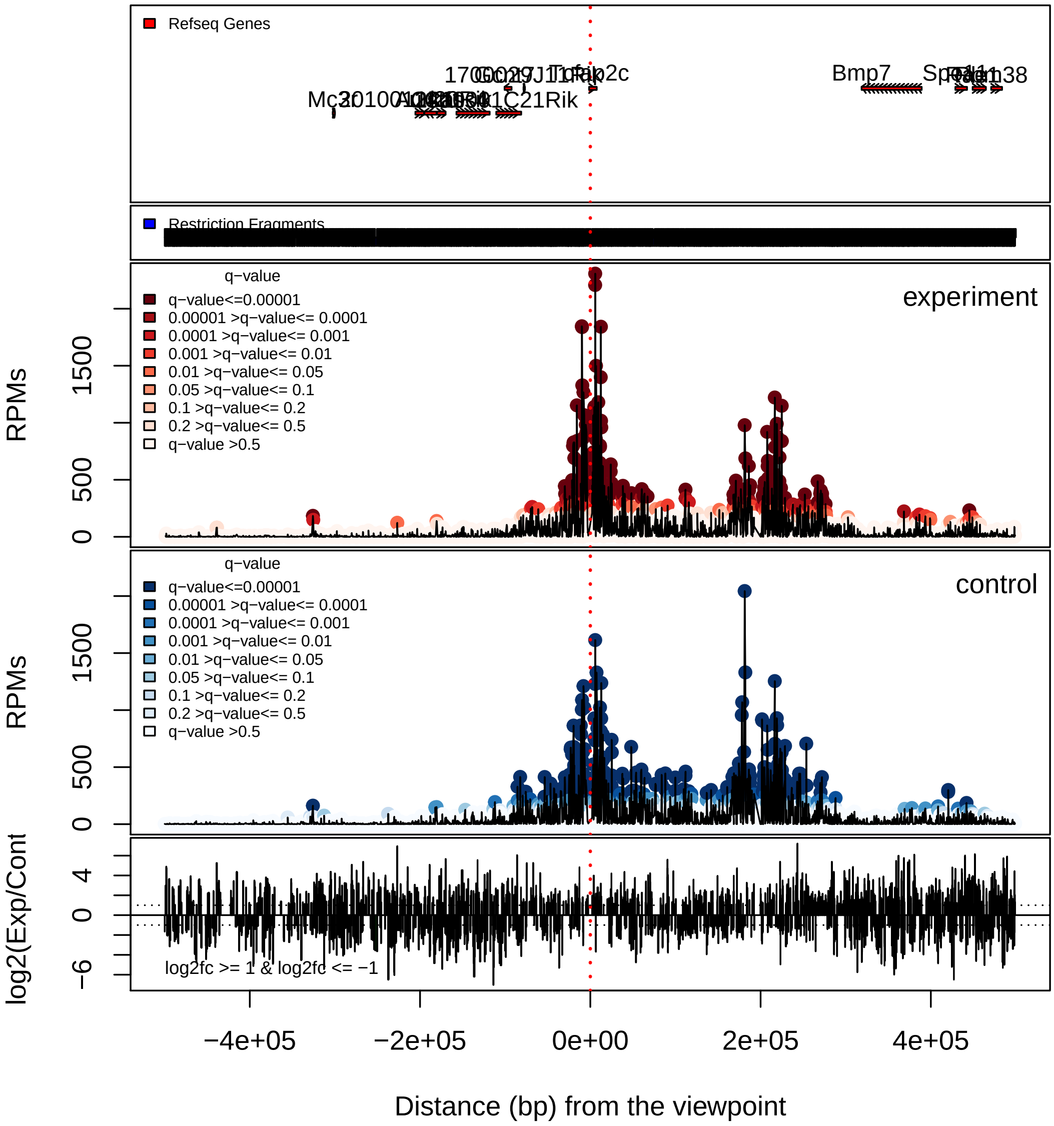

Figure 7: r3Cseq: Overview plot of interactions near the viewpoint.

```
plotInteractionsPerChromosome(my_r3Cseq_obj, "chr2")
```

```
plotOverviewInteractions(my_r3Cseq_obj)
```

Figure 7 shows that *r3Cseq* detects many interactions as soon as the read counts are above a certain threshold. This detection does not seem specific, see e.g. the region approximately 200 kb to the right of the viewpoint. There is one annotated forebrain enhancer with p300 and H3K27ac ChIP-seq signals in this region, which also exhibits DNAseI hypersensitivity in limb and headless embryo tissue ([1, 2]).

2.2 FourCSeq Analysis

We run exactly the same workflow as above for the Myb promoter viewpoint.

```
exptData <- SimpleList(projectPath="FourCSeq_Tfap2c",
                       dataDir="filtered_coverage",
                       fragmentDir="re_fragments",
                       referenceGenomeFile=BSgenome.Mmusculus.UCSC.mm9,
                       reSequence1="CATG",
```

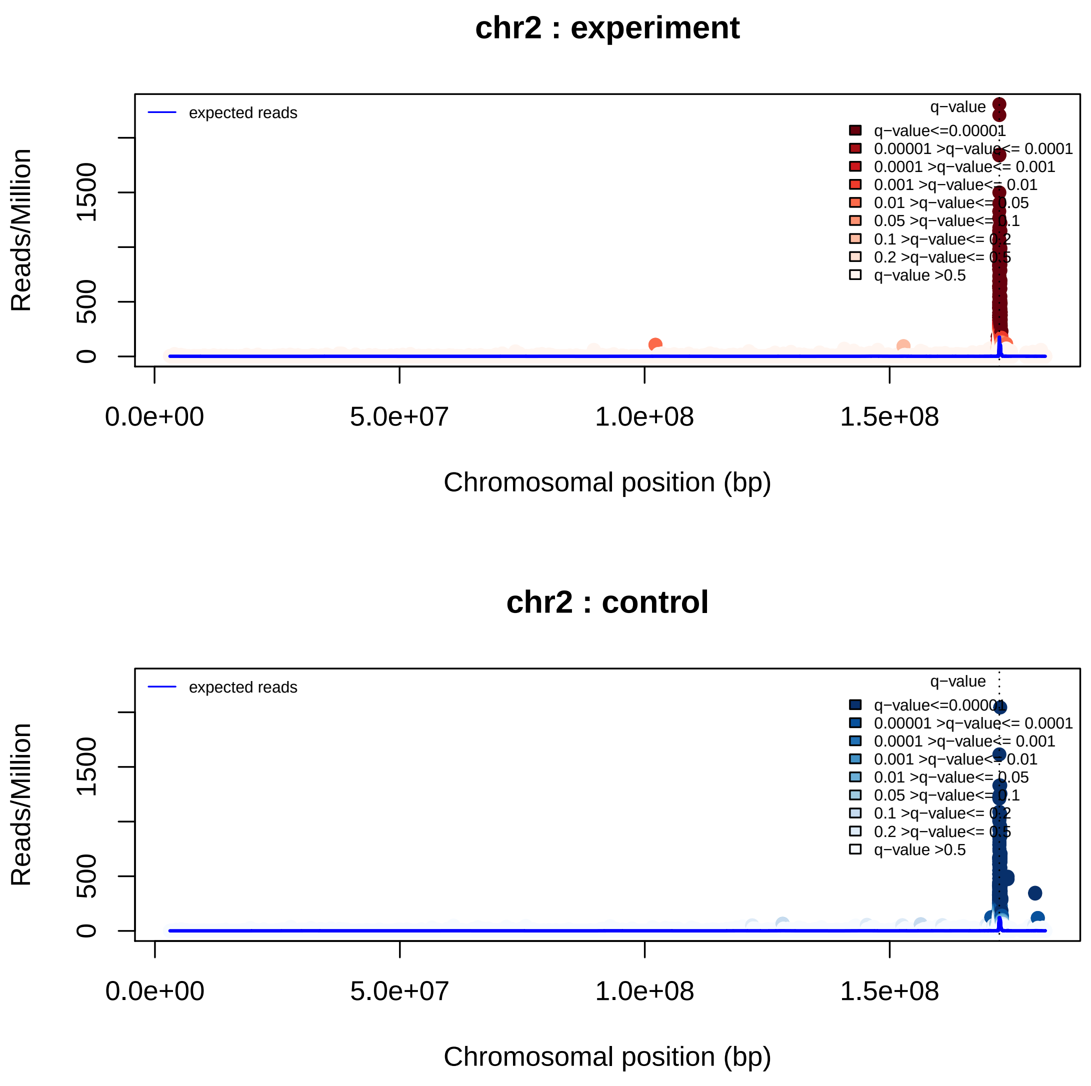

Figure 8: r3Cseq: Overview plot of all interactions on the viewpoint chromosome.

```
                         reSequence2="GATC",
                         primerFile =  "viewpoint_primers.fa",
                         bamFilePath=".")

colData <- DataFrame(viewpoint = c("Tfap2c", "Tfap2c"),
                       condition = c("wt", "wt_MFB"),
                       replicate=c(1,1),
                       bamFile = bamFiles,
                       sequencingPrimer = "first")

fc <- FourC(colData, exptData)

fc <- addFragments(fc, filter=FALSE)
findViewpointFragments(fc)
fc <- addViewpointFrags(fc)
```

After creating a corresponding `FourC` object with the reference fragments we count the number of reads overlapping with fragment ends.

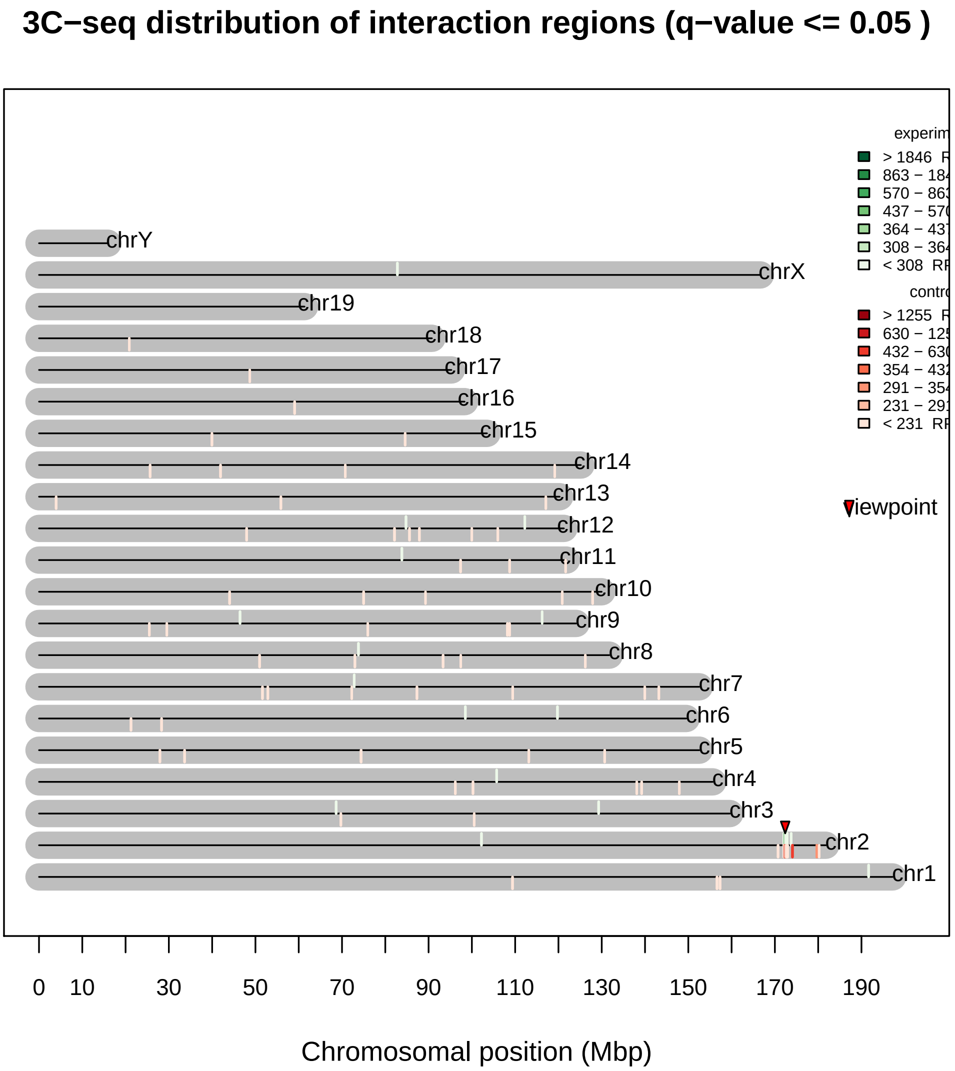

Figure 9: r3Cseq: Overview plot of interactions for all chromosomes.

```
fc <- countFragmentOverlaps(fc, trim=4, minMapq=-1)

## reading bam files
## calculating overlaps

fc <- combineFragEnds(fc)
```

Now we use the getZScores function to detect interactions in the data.

```
fcf <- getZScores(fc, minCount=100)
```

We take a look at the distribution of z-scores, which are stored in the assay "zScores" of the FourC object.

```
zScore <- assay(fcf, "zScore")

for(i in seq_len(ncol(zScore)))
    hist(zScore[,i], breaks=100, main=colnames(zScore)[i])
```

Figure 10 shows that the z-scores are centered close to 0 in both cases.

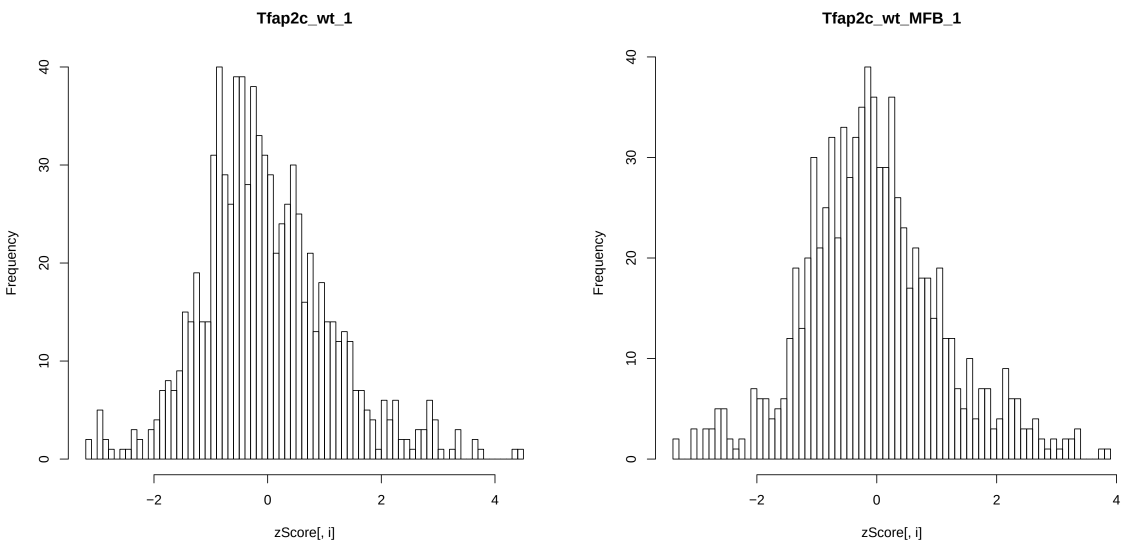

Figure 10: Histograms of z-scores for the different conditions

```
fcf <- addPeaks(fcf, zScoreThresh=2, fdrThresh=0.1)
```

Next we take a look at the fit for the first sample.

```
plotFits(fcf[,1])
```

The points in Figure 11 show the count values for the individual fragments. The red line is the fit and the blue dashed line is the fit plus (z-score threshold)*MAD for the given library, where the z-score threshold has been defined by the call to `addPeaks`. The first plot show the data left of the viewpoint, the second plot right of the viewpoint and for the last plot both sides have been combined by using the absolute distance from the viewpoint.

The results are visualized in the next step.

```
library("TxDb.Mmusculus.UCSC.mm9.knownGene")

fb_enhancer <- GRanges(Rle(c("chr2")),
                       IRanges(start=c(172551999),
                               end=c(172555000)),
                       name=c("FB1"),
                       vp="Tfap2c")

plotZScores(fcf,
            plotWindows = 5e+05,
            txdb=TxDb.Mmusculus.UCSC.mm9.knownGene,
            controls=fb_enhancer)

## [1] "Tfap2c"
```

```
## Successfully plotted results.
```

The Figure 12 shows the results for a 500 kb window around the viewpoint. The fit is shown as green line and the dashed blue lines span the interval of $\pm$ (z-score threshold)*MAD, where the z-score threshold of 2 has been defined by the call to `addPeaks`. Red points represent fragments that have been called as interactions. The position of the annotated forebrain enhancer is shown n the track "VP/TSS" at the bottom.

In these plots we can clearly see that *FourCSeq* specifically detects the interactions with the DNAseI hypersensitive region, which contains an annotated forebrain enhancer ([1, 2]).

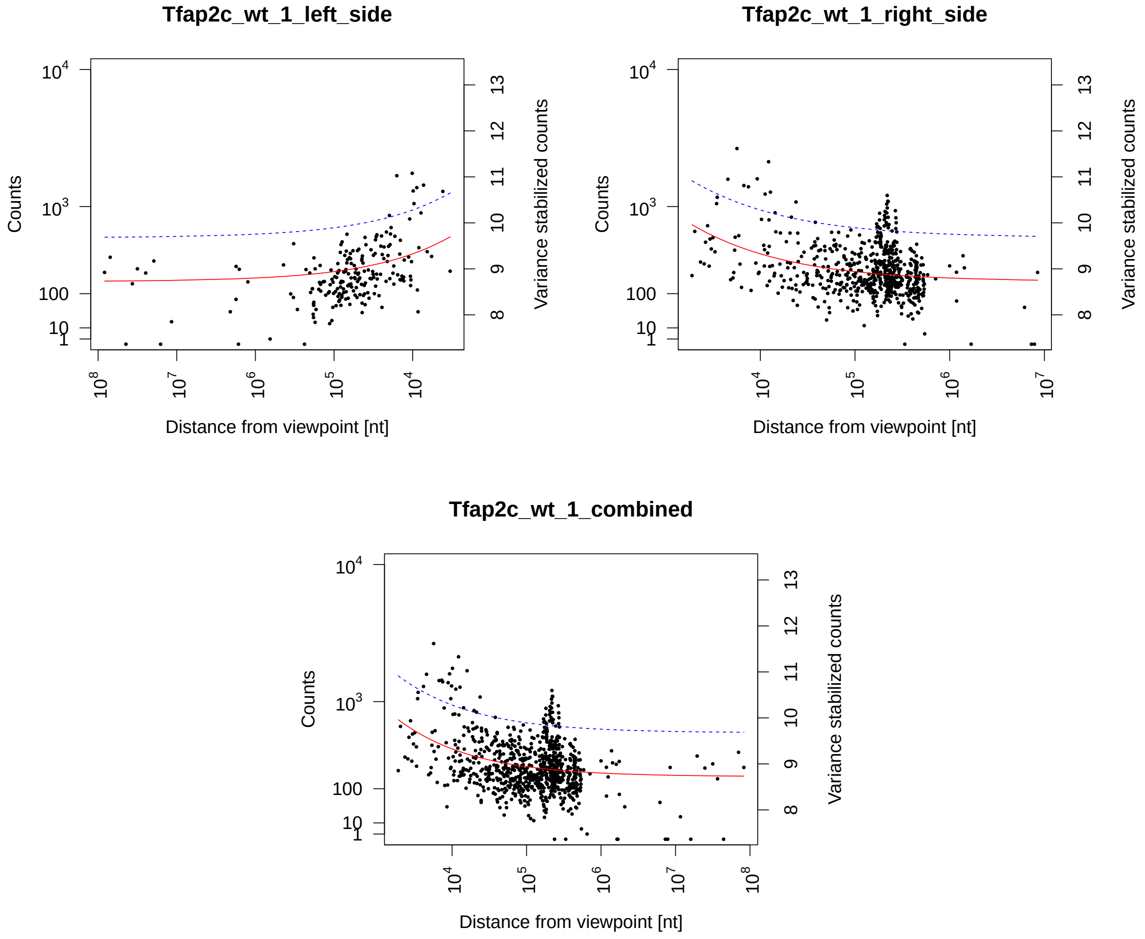

Figure 11: Distance fit on the variance stabilized read counts.

2.3 Result of the comparison

In the case of tha Tfap2c data our *FourCSeq* analysis specifically detects the interaction with the DNAseI hypersensitive region, while the *r3Cseq* analysis detects many unspecific interactions to both sides of the viewpoint. This difference in outcomes appears to be a consequence of FourCSeq using variance stabilized count values, and obtaining a better fit of the 4C seq signal distance dependency.

In general this analysis shows that our *FourCSeq* method is able to specifically detect most likely functional chromatin interactions, while the r3Cseq result in this case lack specificity.

Session Info

```
sessionInfo()

## R version 3.1.2 (2014-10-31)
## Platform: x86_64-unknown-linux-gnu (64-bit)
##
## locale:
##  [1] LC_CTYPE=en_US.UTF-8       LC_NUMERIC=C               LC_TIME=en_US.UTF-8
##  [4] LC_COLLATE=en_US.UTF-8     LC_MONETARY=en_US.UTF-8    LC_MESSAGES=en_US.UTF-8
##  [7] LC_PAPER=en_US.UTF-8       LC_NAME=C                  LC_ADDRESS=C
## [10] LC_TELEPHONE=C             LC_MEASUREMENT=en_US.UTF-8 LC_IDENTIFICATION=C
##
```

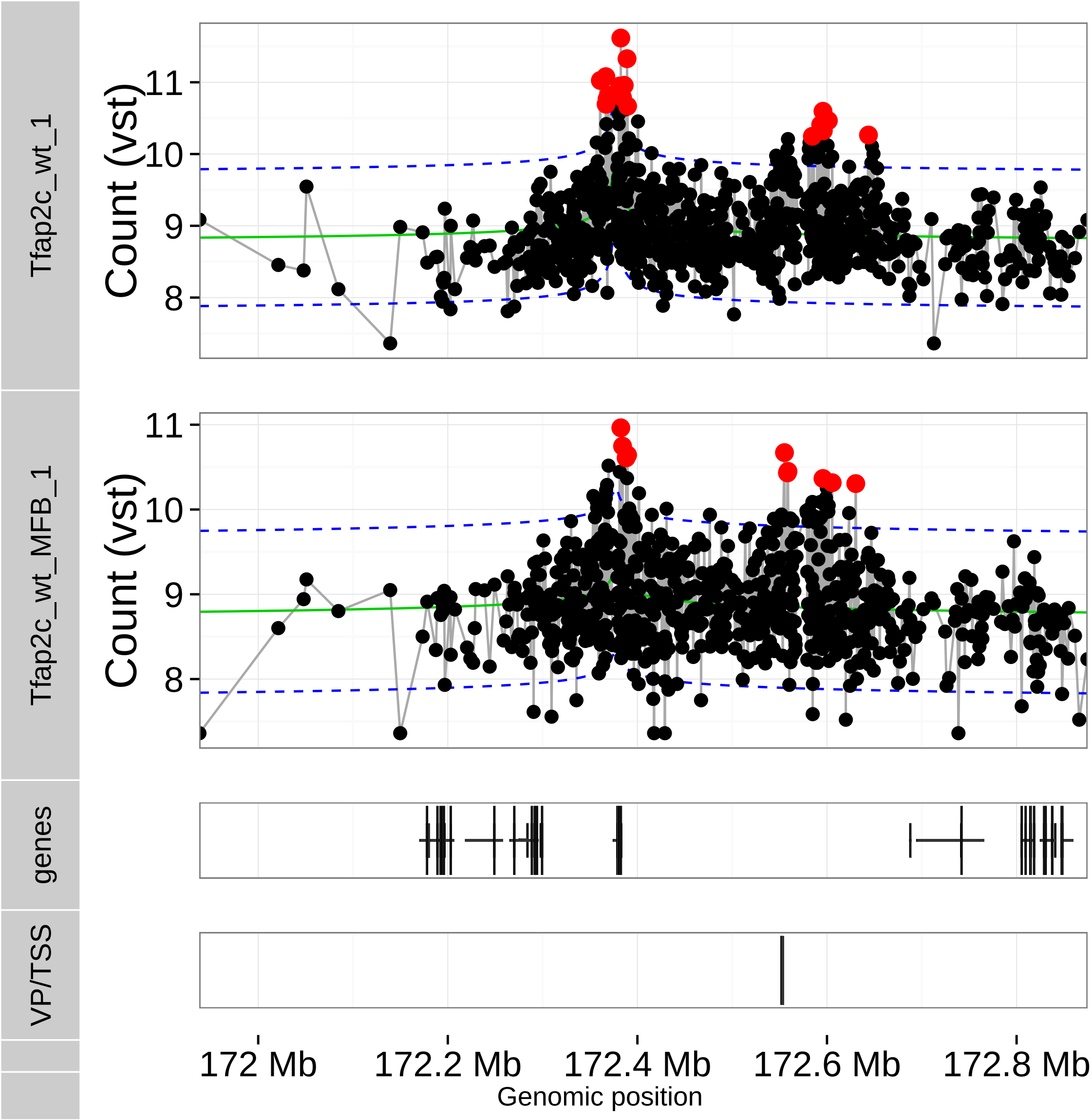

Figure 12: Overview plot of z-scores near to the viewpoint.

```
## attached base packages:
##  [1] splines   stats4    parallel  stats     graphics  grDevices utils     datasets
##  [9] methods   base
##
## other attached packages:
##  [1] TxDb.Mmusculus.UCSC.mm9.knownGene_3.0.0   GenomicFeatures_1.18.7
##  [3] AnnotationDbi_1.28.2                      Biobase_2.26.0
##  [5] BSgenome.Mmusculus.UCSC.mm9.masked_1.3.99 BSgenome.Mmusculus.UCSC.mm9_1.4.0
##  [7] BSgenome_1.34.1                           r3Cseq_1.12.1
##  [9] sqldf_0.4-10                              RSQLite_1.0.0
## [11] DBI_0.3.1                                 gsubfn_0.6-6
## [13] proto_0.3-10                              RColorBrewer_1.1-2
## [15] qvalue_1.43.0                             VGAM_0.9-7
## [17] rtracklayer_1.26.3                        data.table_1.9.4
## [19] Rsamtools_1.18.3                          Biostrings_2.34.1
## [21] XVector_0.6.0                             FourCSeq_1.0.0
## [23] DESeq2_1.6.3                              RcppArmadillo_0.5.100.1.0
## [25] Rcpp_0.11.6                               ggplot2_1.0.1
```

```
## [27] GenomicRanges_1.18.4                        GenomeInfoDb_1.2.5
## [29] IRanges_2.0.1                               S4Vectors_0.4.0
## [31] BiocGenerics_0.12.1                         knitr_1.10
##
## loaded via a namespace (and not attached):
##  [1] acepack_1.3-3.3          annotate_1.44.0          base64enc_0.1-2
##  [4] BatchJobs_1.6            BBmisc_1.9               BiocParallel_1.0.3
##  [7] BiocStyle_1.4.1          biomaRt_2.22.0           biovizBase_1.14.1
## [10] bitops_1.0-6             brew_1.0-6               checkmate_1.5.2
## [13] chron_2.3-45             cluster_2.0.1            codetools_0.2-11
## [16] colorspace_1.2-6         dichromat_2.0-0          digest_0.6.8
## [19] evaluate_0.7             fail_1.2                 fda_2.4.4
## [22] foreach_1.4.2            foreign_0.8-63           formatR_1.2
## [25] Formula_1.2-1            genefilter_1.48.1        geneplotter_1.44.0
## [28] GenomicAlignments_1.2.2  GGally_0.5.0             ggbio_1.14.0
## [31] graph_1.44.1             grid_3.1.2               gridExtra_0.9.1
## [34] gtable_0.1.2             gtools_3.4.2             highr_0.5
## [37] Hmisc_3.15-0             iterators_1.0.7          labeling_0.3
## [40] lattice_0.20-31          latticeExtra_0.6-26      locfit_1.5-9.1
## [43] magrittr_1.5             MASS_7.3-40              Matrix_1.2-0
## [46] munsell_0.4.2            nnet_7.3-9               OrganismDbi_1.8.1
## [49] plyr_1.8.2               RBGL_1.42.0              RCurl_1.95-4.6
## [52] reshape_0.8.5            reshape2_1.4.1           rpart_4.1-9
## [55] scales_0.2.4             sendmailR_1.2-1          stringi_0.4-1
## [58] stringr_1.0.0            survival_2.38-1          tcltk_3.1.2
## [61] tools_3.1.2              VariantAnnotation_1.12.9 XML_3.98-1.1
## [64] xtable_1.7-4             zlibbioc_1.12.0
```

References

[1] Taro Tsujimura, Felix A. Klein, Katja Langenfeld, Juliane Glaser, Wolfgang Huber, and François Spitz. A discrete transition zone organizes the topological and regulatory autonomy of the adjacent *Tfap2c* and *Bmp7* genes. *PLoS Genet*, 11(1):e1004897, 01 2015. URL: http://dx.doi.org/10.1371%2Fjournal.pgen.1004897, doi:10.1371/journal.pgen.1004897.

[2] Axel Visel, Simon Minovitsky, Inna Dubchak, and Len a Pennacchio. VISTA Enhancer Browser–a database of tissue-specific human enhancers. *Nucleic Acids Research*, 35(Database issue):D88–92, January 2007. URL: http://www.pubmedcentral.nih.gov/articlerender.fcgi?artid=1716724&tool=pmcentrez&rendertype=abstract, doi:10.1093/nar/gkl822.
